# Theaflavin-3, 3′-Digallate Suppresses RANKL-Induced Osteoclastogenesis and Attenuates Ovariectomy-Induced Bone Loss in Mice

**DOI:** 10.3389/fphar.2020.00803

**Published:** 2020-06-29

**Authors:** Zexin Ai, Yang’ou Wu, Miao Yu, Jia Li, Shengjiao Li

**Affiliations:** ^1^ Shanghai Engineering Research Center of Tooth Restoration and Regeneration, Department of Oral and Maxillofacial Surgery, School and Hospital of Stomatology, Tongji University, Shanghai, China; ^2^ Shanghai Engineering Research Center of Tooth Restoration and Regeneration, Department of Prosthodontics, School and Hospital of Stomatology, Tongji University, Shanghai, China

**Keywords:** theaflavin-3,3′-digallate, osteoclast, reactive oxygen species, Nrf2, osteoporosis

## Abstract

Theaflavin-3, 3′-digallate (TF3) is extracted from black tea and has strong antioxidant capabilities. The aim of this study was to assess the influences of TF3 on osteoclastogenesis and explore the underlying mechanisms. TF3 efficiently decreased receptor activator of nuclear factor-kappa B ligand (RANKL)-induced osteoclast formation and reactive oxygen species (ROS) generation in a dose-dependent manner. Mechanistically, TF3 reduced ROS generation by activating nuclear factor erythroid 2-related factor 2 (Nrf2) and its downstream heme oxygenase-1 (HO-1) and also inhibited the mitogen-activated protein kinases (MAPK) pathway. Moreover, micro-computed tomography (CT) analysis, hematoxylin and eosin (H&E) staining, and TRAP staining of the femurs of C57BL/6J female mice showed that TF3 markedly attenuated bone loss and osteoclastogenesis in mice. Immunofluorescence staining, 2′,7′-dichlorofluorescein diacetate (DCFH-DA) staining, and measurement of the levels of malonaldehyde (MDA) and superoxide dismutase (SOD) revealed that TF3 increased the expression of Nrf2 and decreased the intracellular ROS level *in vivo*. These findings indicated that TF3 may have the potential to treat osteoporosis and bone diseases related to excessive osteoclastogenesis *via* inhibiting the intracellular ROS level.

## Introduction

Bone, a highly active endocrine organ, undergoes constant remodeling throughout the whole life process. The homeostasis of bone remodeling is maintained in two ways. One is the osteoclastic resorption of old or damaged bone, and the other is the osteoblastic formation of new bone (Feng and McDonald, 2011). Once the balance is broken and bone resorption exceeds bone formation, diseases (e.g., osteoporosis, osteolysis, and periodontitis) may occur (Siddiqui and Partridge, 2016; Monasterio et al., 2019). Therefore, drugs that decrease osteoclastogenesis are clinically significant for ameliorating pathological bone loss.

Osteoclasts are the only cells absorbing bone tissue. They are a kind of polynucleated giant cell derived from mononuclear/macrophages in hematopoietic tissue (Ikeda and Takeshita, 2016). There are two basic factors influencing osteoclastogenesis: macrophage colony-stimulating factor (M-CSF) and receptor activator of nuclear factor-kappa B ligand (RANKL). The former bonds to the receptor c-fms in the osteoclast precursors, increasing their proliferation and survival (Kubatzky et al., 2018). The latter can activate downstream signaling pathways during osteoclastogenesis, including the mitogen-activated protein kinase (MAPK) pathway (Boyle et al., 2003; Takayanagi, 2005). Subsequently, transcription factors of the nuclear factor of activated T-cells (NFATc1) and c-Fos may activate and regulate the differentiation of osteoclast precursors (Asagiri and Takayanagi, 2007).

Reactive oxygen species (ROS) are a kind of highly unstable free radical containing oxygen molecules, such as the superoxide anion (·O2^-^), hydrogen peroxide (H_2_O_2_), and hydroxyl radical (·OH) (Lepetsos et al., 2019). An increasing body of evidence indicates that intracellular ROS play a pivotal role in regulating osteoclastogenesis and bone resorption (Lee et al., 2005; Chen et al., 2019). Moreover, RANKL can elevate the intracellular ROS level in osteoclasts by a signaling cascade involving tumor necrosis factor receptor (TNFR)-associated factor 6 (TRAF6), Rac1, and NADPH oxidase 1 (Nox1) (Lee et al., 2005). Meanwhile, ROS can be taken into account as a signaling messenger during RANKL-induced osteoclastogenesis (Ha et al., 2004). However, the role of ROS in RANKL-stimulated signaling has remained elusive. A certain degree of oxidative stress was indicated to promote the activation of the MAPK (ERK, JNK, p38) pathway (Hyeon et al., 2013). Therefore, to lessen the acceleration of ROS during the formation of osteoclasts, cells typically have protective mechanisms that respond to oxidative stress. One of the methods is to scavenge ROS by nuclear factor erythroid 2-related factor 2/Kelch ECH-associated protein 1 (Nrf2/Keap1) complex (Kanzaki et al., 2017). Nrf2, a transcription factor, participates in the regulation of cytoprotective enzymes. It is expressed in various cells, including osteocytes, osteoblasts, and osteoclasts. Under normal circumstances, Keap1 hitches Nrf2 in the cytoplasm and suppresses its activity. However, when cells are in oxidative stress, Nrf2 may be released from Keap1 and translocated into the nucleus (Kobayashi et al., 2004). Nrf2 then couples with the antioxidant response element (ARE), upregulating the expression of phase II detoxifying enzymes or antioxidant proteins, such as heme oxygenase-1 (HO-1), catalase (CAT), superoxide dismutase (SOD), and glutathione peroxidase 1 (GPx1) (Park et al., 2014; Sun et al., 2015; Tu et al., 2019). Previous studies demonstrated that antioxidants can attenuate osteoclastogenesis and bone loss by improving the expression of cytoprotective enzymes (Lee et al., 2014; Yamaguchi et al., 2018). Thus, these outcomes may assist the suppression of osteoclast formation by inhibiting ROS, which may be a latent strategy for the treatment of bone loss.

Theaflavin-3, 3′-digallate (TF3), one of the major components in black tea, is formed *via* polyreaction of (–)–epigallocatechin gallate (EGCG) and (–)–epicatechin gallate (ECG) (Hu et al., 2017). Moreover, it has been found that TF3 has practical pharmacological activities, such as being anti-inflammatory and antioxidant (Leung et al., 2001; Wu et al., 2017). Recently, a study conducted in Australia indicated that a higher intake of black tea decreased the risk of fracture in older women (Oka et al., 2012). Furthermore, previous studies demonstrated that TF3 inhibited the expression of matrix metalloproteinase 9 (MMP-9) and remedied calvarial osteolysis (Myers et al., 2015; Hu et al., 2017). However, the influences of TF3 on osteoclastogenesis, its activity in preventing osteoporosis, and the possible mechanisms have remained obscure.

With respect to the crucial role of ROS in osteoclast formation and the antioxidant activity of TF3, in the present study, we assumed that TF3 inhibits osteoclastogenesis through activating the Nrf2/Keap1 pathway, as well as inhibiting the MAPK pathway. In the current study, we examined the effects of TF3 both *in vitro* and *in vivo* as well as the potential mechanisms of TF3 in osteoclast formation.

## Materials and Methods

### Materials and Reagents

**Figure 1:**
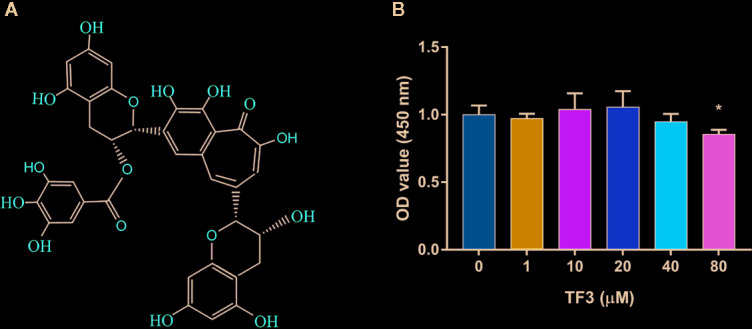
Effects of TF3 on cell viability. **(A)** The chemical formula of TF3. **(B)** Cell viability of BMMs treated with various concentrations of TF3 (0, 1, 10, 20, 40, or 80 μM) for 5 days. n = 3, ^*^ *P* < 0.05 compared with control group (without TF3 treatment).

TF3 (purity>98%, **Figure 1A**), purchased from Shanghai Fulong Biotechnology Co., Ltd. (Shanghai, China) and analyzed by high-performance liquid chromatography (HPLC), was dissolved in pure ethyl alcohol, which was stored at -20°C with no direct light; the final concentration of absolute ethyl alcohol was lower than 0.1% after dilution. Alpha modification of Eagle’s minimum essential medium (α-MEM, penicillin–streptomycin solution, and fetal bovine serum (FBS) were acquired from HyClone Laboratories Inc. (Logan, UT, USA). Cell counting Kit-8 (CCK-8), Red Blood Cell Lysis Buffer, Nuclear and Cytoplasmic Protein Extraction kit, bicinchoninic acid (BCA) assay kit, 4′,6-Diamidino-2′-phenylindole dihydrochloride (DAPI), and Triton X-100 were purchased from Beyotime Biotechnology (Shanghai, China). Rhodamine-conjugated Phalloidin was obtained from Yeasen Biotech Co., Ltd. (Shanghai, China). M-CSF and RANKL were provided by PeproTech (Rocky Hill, NJ, USA). TRAP staining kit and 2′,7′-dichlorodihydrofluorescein diacetate (DCFH-DA) were purchased from Sigma-Aldrich (St. Louis, MO, USA). Trizol reagent and Reverse Transcription Kit were purchased from TaKaRa (Otsu, Japan). Specific primary antibodies such as β-actin, Lamin B, Nrf2, HO-1, ERK, phosphor-ERK, JNK, phospho-JNK, p38, phosphor-p38, and CTSK were provided by Cell Signaling Technology (Danvers, MA, USA). Malondialdehyde (MDA) assay kit and SOD assay kit were purchased from Nanjing Jiancheng Bioengineering Institute (Nanjing, China).

### Cell Culture and Differentiation

Bone marrow macrophages (BMMs) were isolated from the tibias and femurs of 8-week-old C57BL/6 mice and cultured in α-MEM, containing 10% FBS, 100 U/ml of penicillin, and 100 μg/ml of streptomycin for 48 h. The cells were maintained in a humidified incubator with a 95% air/5% CO_2_ atmosphere at 37°C. We collected the suspension of non-adherent cells and purified using Red Blood Cell Lysis Buffer. Next, BMMs were planked in cell culture plates, which were treated with 50 ng/ml M-CSF and different concentrations of TF3 (0, 1, and 10 μM) for 3 days. After that, BMMs were induced with M-CSF (50 ng/ml) and RANKL (100ng/ml) in the presence of TF3 (0, 1, and 10 μM). The fluid was changed every two days for three times. All *in vitro* experiments were categorized into four groups: NC, negative control group, treated with only 50 ng/ml M-CSF; C, control group, stimulated with 50 ng/ml M-CSF and 100 ng/ml RANKL; L, low-concentration TF3 group, treated with 50 ng/ml M-CSF, 100 ng/ml RANKL, and 1 μM TF3; H, high-concentration TF3 group, treated with 50 ng/ml M-CSF, 100 ng/ml RANKL, and 10 μM TF3.

### Cell Viability Assay

BMMs were seeded into 96-well plates with a density of 2×10^3^ cells/well overnight. Cells were cultured in M-CSF (50 ng/ml) and various concentrations of TF3 (0, 1, 10, 20, 40, and 80 μM) for 5 days, respectively. The cells were then rinsed with phosphate-buffered saline (PBS), and the culture medium was replaced with a fresh culture medium containing 10 μl CCK-8 and 90 μl α-MEM. After incubation for 2 h at 37°C in the dark, the optical density (OD) was measured by a microplate reader at the wavelength of 450 nm.

### *In Vitro* Osteoclastogenesis Assay

BMMs were seeded into a 24-well plate at a density of 6×10^5^ cells/well and induced to osteoclasts as mentioned above. After 5 days, the medium was discarded and the cells were lightly washed thrice with PBS. Cells were then fixed with 4% paraformaldehyde at room temperature for 30 min and washed thrice with PBS for 5 min. Next, cells were stained using a TRAP staining kit at 37°C for 15 min in the dark incubator. TRAP-positive multinucleated cells containing at least three nuclei were taken into account as osteoclasts.

### F-Actin Ring Formation Assay

BMMs were seeded into a 24-well plate at a density of 6×10^5^ cells/well and induced to mature osteoclasts, as mentioned earlier. After the formation of osteoclasts, the medium was replaced with PBS to wash the cells three times. The cells were fixed as mentioned above, then permeabilized by 0.1% Triton X-100 for 15 min, and subsequently stained with rhodamine-conjugated phalloidin in the dark for 40 min. Afterward, the cells were irrigated thrice with PBS and counterstained with DAPI for 10 min to show nuclei. F-actin rings were photographed with a fluorescent microscope.

### Intracellular ROS Assay

We used DCFH-DA, a fluorescent probe with the ability to permeate cells, to detect the levels of intercellular ROS. BMMs were seeded into a 24-well plate at a density of 2×10^4^ cells/well overnight. Cells were treated with 50 ng/ml M-CSF for 3 days and then treated with or without 100 ng/ml RANKL, as well as with different concentrations of TF3 (0, 1, and 10 μM) for 4 h. Then the cells were flushed with PBS and incubated with α-MEM containing 10 μM DCFH-DA for 30 min in the dark. The cells were re-washed thrice with PBS, and the fluorescence signal was detected at 485 nm excitation and 538 nm emission settings under a fluorescence microscope.

### Real-Time Quantitative Polymerase Chain Reaction (RT-qPCR)

BMMs were seeded into a six-well plate with a density of 3×10^6^ cells/well and cultured to osteoclasts as described above. Total RNA was extracted with Trizol reagent according to the manufacture’s instruction. We used 1 μg RNA to synthesize cDNA with a Reverse Transcription Kit. RT-qPCR was performed using an SYBR Green PCR kit. Each reaction was run for 40 cycles of 95°C for 15 s, 60°C as the annealing temperature for 30 s, and an extension step for 30 s at 72°C. Glyceraldehyde 3 phosphate dehydrogenase (*GAPDH)* served as a reference gene, and the mouse-specific primer sequences for *GAPDH*, *TRAP*, *MMP-9*, *CTSK*, *Nrf2*, *HO-1*, *CAT, SOD,* and *GPx1* are shown in **Supplementary Table S1**. Data were analyzed with LightCycler96 software.

### Western Blot Analysis

For Western blotting, BMMs were treated as explained above. The cells were lysed by Nuclear and Cytoplasmic Protein Extraction Kit and RIPA lysis buffer, respectively. The protein concentrations were assessed with a BCA kit. Proteins were separated using sodium dodecyl sulfate-polyacrylamide gel electrophoresis (SDS-PAGE) and then transferred onto polyvinylidene fluoride (PVDF) membranes. Next, the membranes were blocked with 5% bovine serum albumin (BSA) for 2 h and incubated with primary antibodies at 4 °C overnight. After washing, they were incubated with secondary antibodies for 2 h. The membranes were washed in TBST for 10 min, and protein bands were detected with a chemiluminescent reagent kit. Data were analyzed by Image J software (National Institutes of Health, Bethesda, MD, USA).

### *In Vivo* Animal Model

This study was carried out in accordance with the recommendations of the Animal Ethics Committee of Tongji University, and all animal procedures were approved by the Animal Ethics Committee of Tongji University (Approval No. TJLAC-018-035; Shanghai, China). Animals were reared in ventilated cages with a 12-h light/dark cycle. The mice were fed on standard water and diet ad libitum. Herein, 24 8-week-old C57BL/6J female mice were randomly divided into four groups: sham-operated group, ovariectomized (OVX) group, OVX + low dose TF3 (1 mg/kg) group, and OVX + high dose TF3 (10 mg/kg) group. All mice were injected intraperitoneally one week after the surgery. Mice in sham-operated and OVX groups were injected with the same volume of PBS containing 1% absolute ethyl alcohol as control. Mice were injected three times every week. Animals were sacrificed after three months, and the bilateral lower limbs of each mouse were dissected and fixed in 4% paraformaldehyde.

### Micro-Computed Tomography (CT) and Histological Assessments

Excess soft tissue was removed from the femurs, then they were prepared to undergo micro-CT scans. We selected 50 slices as a region of interest (ROI), which was under the growth plate by the height of 100 slices. Cancellous bone parameters within the ROI were analyzed. After scanning, femurs were decalcified and embedded in paraffin wax for sectioning, and sections with a thickness of 4 μm were cut. Hematoxylin and eosin (H&E) and TRAP staining methods were then performed. Slices were observed by light microscope.

### Measurement of Levels of MDA and SOD

Blood samples were collected from the abdominal aorta of mice. The samples were deposited at room temperature for 30 min and then centrifuged at 4 °C for 15 min. We collected the supernatant, which was the serum of mice. Next, the concentrations of serum were assessed with a BCA kit. The levels of MDA and SOD in serum were determined by MDA assay kit and SOD assay kit according to the manufacturer’s instructions, respectively. The OD value was measured by a microplate reader at wavelengths of 530 nm and 450 nm, respectively.

### Fluorescent Staining

The immunofluorescence staining was performed with paraffined sections of femurs. Rabbit anti-mouse Nrf2 antibody was diluted with PBS at 1:300, and the sections were incubated overnight at 4°C. The slides were rinsed with PBS and incubated with goat anti-rabbit IgG immunofluorescence secondary antibody (dilution, 1:1000) at 37°C for 1 h. The slides were washed thrice with PBS, followed by DAPI counterstaining. For *in vivo* ROS detection, mice were intravenously injected with 200μL DCFH-DA at 25 mg/kg and killed after 24 h, and cryosections were prepared according to previously described protocol (Chen et al., 2019). Sections were stained with rabbit anti-mouse CTSK antibody (dilution, 1:300) and goat anti-rabbit IgG immunofluorescence secondary antibody, which was followed by DAPI counterstaining. Images were obtained by using a fluorescence microscope.

### Statistical Analysis

All *in vitro* experiments were repeated three times, and *in vivo* data were from six different mice. Data were statistically analyzed by SPSS 20.0 software (IBM, Armonk, NY, USA). All statistical graphs were drawn with GraphPad Prism 7.0 software (GraphPad Software Inc., San Diego, CA, USA). All data were presented as mean ± standard error of the mean (SEM). The statistical differences among groups were determined by one-way analysis of variance (ANOVA). *P* < 0.05 was considered statistically significant.

## Results

### Effects of TF3 on Cell Viability

To detect the cytotoxicity of TF3 (**Figure 1A**), we cultured BMMs with different concentrations (0-80 μM) of TF3 for 5 days. As shown in **Figure 1B**, TF3 did not have cytotoxicity at levels of less than 80 μM compared with the control group. Based on the results achieved, we selected 1 μM and 10 μM as target concentrations for subsequent experiments.

### TF3 Inhibited RANKL-Induced Osteoclastogenesis and F-Actin Ring Formation In Vitro

**Figure 2:**
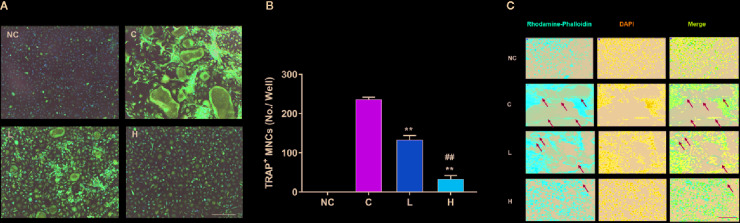
Effects of TF3 on RANKL-induced osteoclastogenesis and F-actin ring formation *in vitro*. **(A)** BMMs were treated with M-CSF and RANKL containing various concentrations of TF3 (0, 1, 10 μM) for 5 days, and TRAP staining was then performed. **(B)** TRAP^+^ multinucleated cells with more than three nuclei were counted as osteoclasts. **(C)** Representative images showing the F-actin formation in osteoclasts treated with TF3. n = 3, ^**^ *P* < 0.01 compared with C group. ^##^ *P* < 0.01 compared with L group. Scale bar = 100 µm.

To indicate the effects of TF3 on osteoclastogenesis, we induced BMMs to osteoclast. The negative control group without RANKL and TF3 had no osteoclast formation. The control group, stimulated by M-CSF and RANKL, exhibited a mass of TRAP-positive and multinucleated osteoclasts. It was also noted that the formation of osteoclasts was significantly suppressed by TF3 (**Figure 2A**). Furthermore, counting of TRAP-positive multinucleated osteoclasts indicated that osteoclast differentiation was inhibited by TF3 in a dose-dependent manner (**Figure 2B**).

F-actin rings are dynamic and unique cytoskeletal structures that are a mark of the resorbing osteoclast (Cappariello et al., 2014; Thummuri et al., 2017). RANKL facilitates the formation of F-actin rings. Therefore, we examined the effects of TF3 on the formation of F-actin rings. The results of rhodamine-conjugated phalloidin staining showed that RANKL increased the formation of F-actin rings compared with the negative control group; however, TF3 suppressed the formation of F-actin under RANKL-induced condition (**Figure 2C**). The size and number of F-actin rings were decreased as the concentration of TF3 increased.

### TF3 Suppressed RANKL-Induced ROS Generation by Enhancing the Expression of Antioxidant Genes

**Figure 3:**
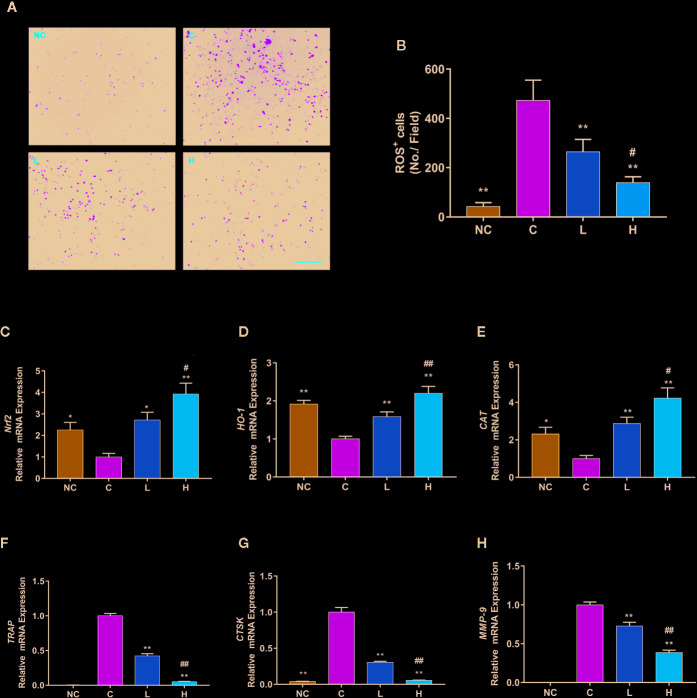
TF3 attenuates RANKL-induced ROS generation *in vitro*. **(A)** Representative images of RANKL-induced ROS generation detected by DCFH-DA in BMMs with different concentrations of TF3. **(B)** Quantification of the number of ROS-positive cells per field. **(C-E)** RT-qPCR analysis showed the expression levels of antioxidant genes in BMMs. **(F–H)** The mRNA expression of osteoclast-specific marker genes. n = 3, ^*^ *P* < 0.05, ^**^ *P* < 0.01 compared with C group. ^#^ *P* < 0.05, ^##^ *P* < 0.01 compared with L group. Scale bar = 100 µm.

ROS have been found to be a vital mediator regulating the differentiation of RANKL-stimulated osteoclast (Lu et al., 2015). To indicate whether TF3 can affect intracellular ROS generation, we used a cell-permeable fluorescent probe, DCFH-DA, which is de-esterified endocellularly and oxidizes to a highly fluorescent 2′,7′-dichlorofluorescein (DCF). The results revealed that RANKL stimulation dramatically increased intracellular ROS generation compared with the without RANKL stimulation groups. Moreover, the inhibition of TF3 was dose-dependent (**Figures 3A, B**). Due to the antioxidant activity of TF3, we speculated whether TF3 could inhibit intracellular ROS generation by activating Nrf2. Next, to illustrate the conjecture, we detected the expression levels of *Nrf2* and *Nrf2*-mediated downstream genes. The results of RT-qPCR indicated that TF3 upregulated the expression levels of *Nrf2, HO-1*, and *CAT* in a dose-dependent manner (**Figures 3C–E**). The expression levels of *SOD* and *GPx1* were also upregulated by TF3 (**Supplementary Figure S1**). We also detected the mRNA expression of osteoclast-specific marker genes. The RANKL-induced osteoclastogenesis group showed significantly upregulated osteoclast-specific marker genes, including *TRAP*, *CTSK*, and *MMP-9*. The corresponding expressions of levels in groups containing TF3 were downregulated dose-dependently (**Figures 3F–H**). Therefore, it could be concluded that TF3 suppressed osteoclastogenesis by enhancing antioxidant enzymes as well as decreasing intracellular ROS generation.

### TF3 Activated the Nrf2/HO-1 Signaling Pathway and Inhibited RANKL-Induced Phosphorylation of the MAPK Pathway

**Figure 4:**
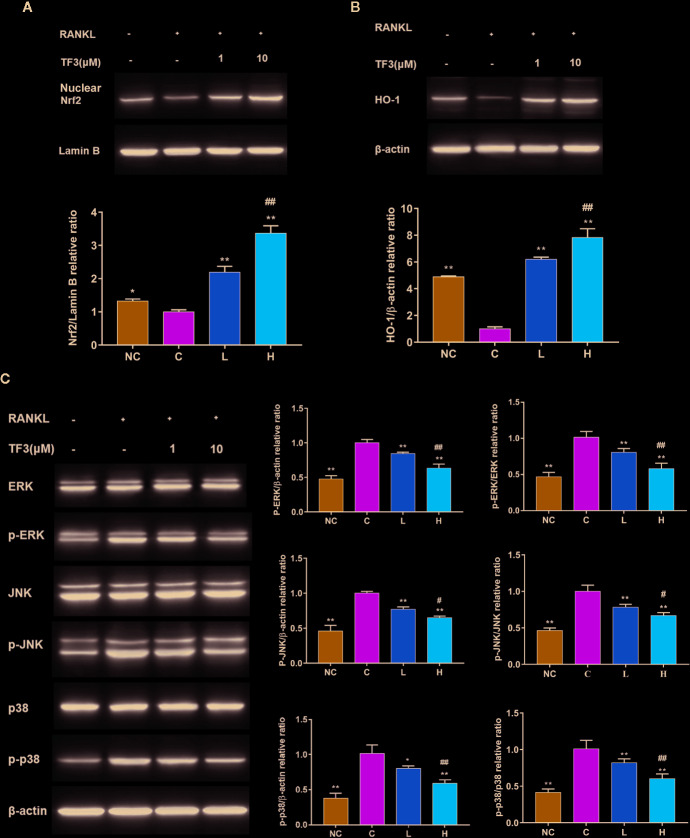
TF3 activated the Nrf2/HO-1 signal pathway and inhibited RANKL-induced phosphorylation of the MAPK pathway. **(A)** The effects of TF3 on the expression of Nrf2 in nucleus. **(B)** The influences of TF3 on the expression of HO-1. **(C)** The effects of TF3 on the MAPK pathway. n = 3, *P < 0.05, **P < 0.01 compared with C group. ^#^ *P* < 0.05, ^##^ *P* < 0.01 compared with L group.

We further studied the protein expression levels of Nrf2 and Nrf2-induced antioxidant protein HO-1. We observed dose-dependently incremental nuclear translocation of Nrf2 in the TF3 treatment groups (**Figure 4A**). TF3 also increased the expression level of Nrf2-mediated cytoprotective protein HO-1 (**Figure 4B**). It can be inferred from these results that TF3 alleviates the oxidative stress of osteoclast by upregulating the Nrf2/HO-1 signaling pathway.

The MAPK pathway is one of the most important pathways in the process of osteoclast formation. Meanwhile, ROS may play a substantial role in the activation of MAPK pathways. Thus, we examined the protein level of MAPK pathways in the case of elevated antioxidant enzymes. The results showed that phospho-ERK, phospho-JNK, and phospho-p38 were markedly suppressed by TF3 treatment compared with total protein, respectively (**Figure 4C**). Collectively, TF3 facilitated the activation of the Nrf2/HO-1 signaling pathway and inhibition of MAPK phosphorylation.

### TF3 Reduced OVX-Induced Bone Loss and Osteoclast Formation In Vivo

**Figure 5:**
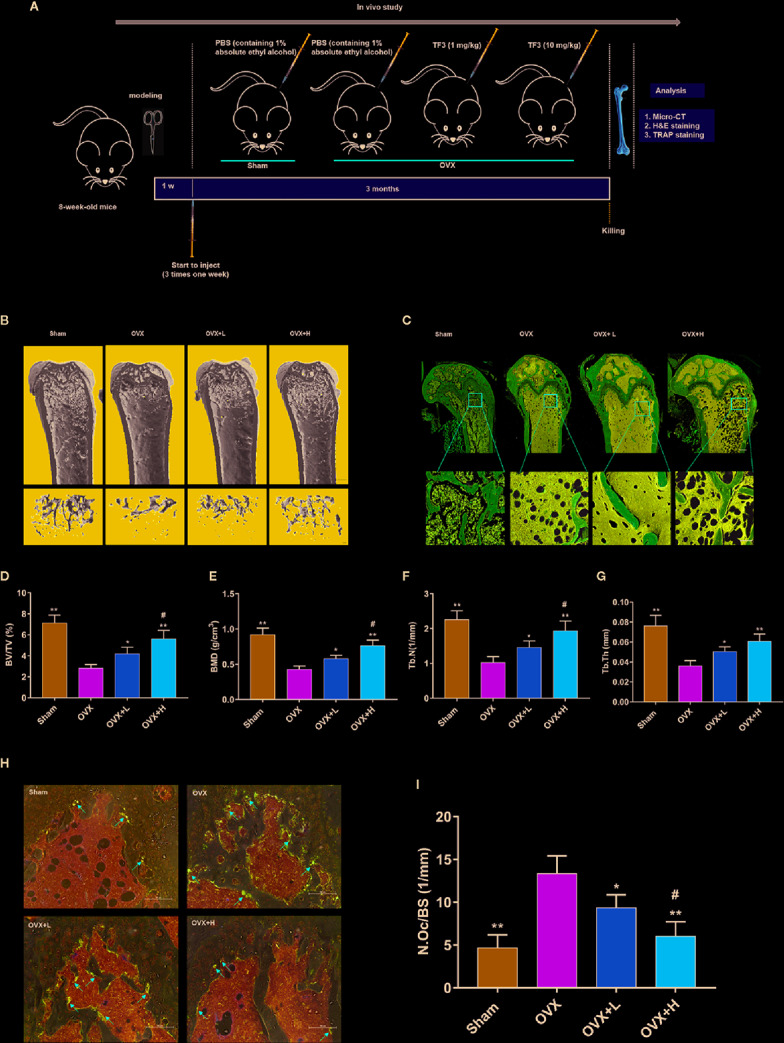
TF3 prevented OVX-induced bone loss and osteoclast formation *in vivo*. **(A)** Schematic illustration of the establishment of the OVX mice model and the experimental design. **(B)** Representative micro-CT images displayed the effects of TF3 on bone loss. Scale bar = 1 mm. **(C)** Representative images of H&E staining of decalcified bone sections. Scale bar = 100 μm. **(D–G)** Quantitative analyses of parameters related to bone microstructure, including BV/TV, BMD, Tb.N, and Tb.Th. **(H)** Representative images of TRAP staining of decalcified bone sections. Scale bar = 100 μm. **(I)** Quantitative analyses of N.Oc/BS. n = 6, ^*^ *P* < 0.05, ^**^ *P* < 0.01 compared with OVX group. ^#^ *P* < 0.05 compared with OVX + L group.

We assessed whether TF3 could decrease bone loss and osteoclast formation *in vivo* (**Figure 5A**). We examined the influences of TF3 on an OVX mouse model. Micro-CT and H&E staining showed that bone loss was remarkably found in the OVX group compared with the sham-operated group. The loss of trabeculae was attenuated in low-dose and high-dose groups in a dose-dependent manner (**Figures 5B, C**). Quantitative analyses of trabecular bone parameters led to the same conclusions (**Figures 5D–G**).

To further assess whether osteoclasts could be involved in the inhibitory effect of TF3 on bone loss caused by OVX, TRAP staining was used to count the number of osteoclasts. TRAP-positive multinucleated osteoclasts in the OVX group were significantly higher than those in the sham-operated, low-dose, and high-dose groups (**Figure 5H**). Furthermore, the number of osteoclasts in the high-dose group was lower than that in the low dose group. Histomorphometric analysis of the number of osteoclasts confirmed that TF3 treatment attenuated OVX-induced bone loss and reduced the number of osteoclasts as well (**Figure 5I**). The above-mentioned data suggested that OVX-caused bone loss was effectively prevented by TF3 in a dose-dependent manner *in vivo*. Moreover, we further confirmed that TF3 (1 mg/kg and 10 mg/kg) did not lead to tissue damage *in vivo*. After injection of TF3 for 3 months, no obvious histological changes were found in H&E staining in the heart, liver, spleen, lung, and kidney of mice (**Supplementary Figure S2**).

### TF3 Remitted Oxidative Stress and Increased the Expression of Nrf2 In Vivo.

**Figure 6:**
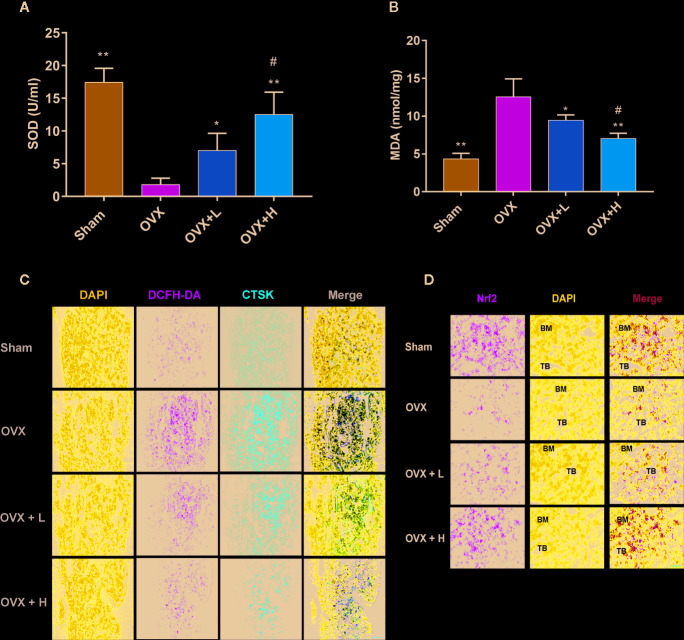
TF3 remitted oxidative stress and increased the expression of Nrf2 *in vivo*. **(A)** Serum SOD level in mice. **(B)** Serum MDA level in mice. **(C)** DCFH-DA staining and immunofluorescence staining of CTSK showed the intracellular ROS level in femur. **(D)** The immunofluorescence staining displayed the expression of Nrf2 in femur. n = 6. ^*^ *P* < 0.05, ^**^ *P* < 0.01 compared with OVX group. ^#^ *P* < 0.05 compared with OVX + L group. Scale bar = 100 μm.

To examine the effects of TF3 on oxidative stress caused by OVX, we detected two indicators of oxidative stress. The serum MDA level in mice treated with TF3 was lower than that in OVX mice and decreased with an increase in TF3 dose (**Figure 6A**). The activities of SOD in the TF3 treatment groups were significantly higher than those in the OVX group and were enhanced with an increase in TF3 dose (**Figure 6B**). The results indicated that TF3 remitted oxidative stress caused by OVX. Therefore, we examined the expression of Nrf2 in femur. Immunofluorescence staining revealed that TF3 enhanced the expression of Nrf2 compared with the OVX group. Besides, we detected the ROS level in osteoclasts marked by CTSK in femur, and fluorescent staining showed that TF3 reduced the level of ROS in osteoclasts of femur compared with the OVX group (**Figures 6C, D**). Accordingly, TF3 reduced ROS generation *in vivo* by remitting oxidative stress and enhancing the expression of Nrf2.

## Discussion

Osteoporosis is a systemic bone disease associated with a decrease in bone mass, destruction of bone microarchitecture, and increased risk of fracture (Compston et al., 2019). It is also closely associated with hyperactive osteoclast activity (Binder et al., 2009). Thus, intervention in osteoclast is highly significant for osteoporosis treatment. In the present study, we assessed the effects of TF3 on osteoclastogenesis and osteoporosis. Our results elucidated that TF3 inhibited osteoclastogenesis by reducing the ROS level and decreased bone loss during osteoporosis caused by OVX.

To determine the effects of TF3 on osteoclast formation and differentiation, we performed osteoclastogenesis assay and F-actin ring formation assay *in vitro*. The results showed that TF3 markedly reduced the quantity and size of osteoclasts and F-actin, which indicated that TF3 may be a new therapy for bone disease caused by overactive osteoclasts. Osteoclast formation is a complicated process influenced by a variety of factors, such as hormones, cytokines, and transcription factors. Recently, ROS have been identified as a vital mediator regulating RANKL-stimulated osteoclast differentiation (Lu et al., 2015). Thus, because of the antioxidant activity of TF3, we studied the mechanisms of this inhibitory effect of osteoclast formation, concentrating on the intracellular ROS level, Nrf2 signaling pathway, and MAPK pathway.

After stimulation with RANKL, TF3 suppressed the intracellular ROS level elevated by RANKL. The intracellular ROS level is maintained by the equilibrium between the rate of generation and the rate of elimination. Thus, we speculated that the decreased intracellular ROS level might be due to an enhanced ability of cells to scavenge ROS. Afterward, we detected several ROS scavengers, including *CAT*, *HO-1, SOD,* and *GPx1*. These genes were significantly upregulated by TF3 treatment in the present study. Previous research revealed that H_2_O_2_ is the major ROS resulting in osteoclast formation and activity (Bax et al., 1992; Fraser et al., 1996). Accordingly, *CAT* is an enzyme that decomposes H_2_O_2_ into oxygen and water (Hao and Liu, 2019). *HO-1* plays a vital role in catalyzing heme liberated by oxidants (Hayashi et al., 1999).

Furthermore, we investigated the up-regulatory mechanism of these antioxidant genes. In fact, Nrf2, a vital transcriptional factor, exists widely in a variety of tissues (Yang et al., 2013) and induces the expression of numerous cytoprotective enzymes in response to oxidative stress, including *HO-1*, *CAT*, etc. (Bae et al., 2013; Cheleschi et al., 2017). Kanzaki et al. (2013) reported that osteoclasts could scavenge redundant ROS through the Nrf2 signaling pathway. In the present research, RANKL decreased the expression of *Nrf2* to strengthen ROS signaling, while TF3 upregulated the levels of Nrf2 to scavenge ROS. Additionally, we examined the protein levels of Nrf2 and HO-1. Nrf2 is translocated from cytoplasm into the nucleus before it starts to work. Therefore, we detected the protein level of nuclear Nrf2. The results of Western blotting suggested that the level of nuclear Nrf2 in the control group was lowest and that TF3 upregulated the translocation of Nrf2 in a dose-dependent manner. The expression of HO-1 was consistent with Nrf2. At the same time, the results also indicated that the expression changes of antioxidant genes caused homologous changes in proteins. Hence, the mechanism of TF3-decreased ROS level may upregulate CAT and HO-1 expression through Nrf2.

An increasing amount of evidence indicates that activation of the MAPK pathway, containing three major family members (ERKs, JNKs, and p38), plays a significant role in RANKL-stimulated osteoclast differentiation and formation (Tanaka et al., 2006; Liao et al., 2019). ERK is vital for the survival of osteoclast, and JNK and p38 are also crucial for the differentiation and function of osteoclast (Lee et al., 2002; Koga et al., 2019). Moreover, ROS, as a second messenger, can activate the MAPK pathway (Lee et al., 2005). Therefore, based on the above-mentioned findings, we assessed whether TF3 suppressed osteoclastogenesis by inhibiting the MAPK pathway. Our results revealed that TF3 exerted significantly suppressive effects on phosphorylation of ERK, JNK, and p38 compared with the control group. A previous study reported that the MAPK pathway can enhance the translocation of Nrf2 into the cell nucleus under oxidative stress in cardiomyocytes, thereby increasing the level of antioxidative enzymes (e.g., HO-1) (Zhao et al., 2016). However, the relationship between Nrf2 and MAPK in osteoclasts needs to be further assessed in future studies.

Based on the *in vitro* results, we further studied the therapeutic effects of TF3 on osteoporosis *in vivo* using a classical OVX mouse model. In the current study, TF3 showed a protective effect on bone loss and reduced osteoclastogenesis, as verified by micro-CT, H&E staining, and TRAP staining. Our results were consistent with previously reported findings that TF3 can suppress osteoclastogenesis (Nishikawa et al., 2015; Hu et al., 2017) and OVX-caused bone loss. However, with respect to the pharmacological activity of TF3, the optimal dose for clinical treatment of osteoporosis may be worthy of further exploration. MDA is formed when oxygen free radicals attack unsaturated fatty acids in cell membranes, and it represents the degree of cell damage (Qin et al., 2019). SOD is one of the critical antioxidant enzymes, and the activity of SOD reflects the ability to scavenge oxygen free radicals (Zelko et al., 2002). TF3 can remit the oxidative stress by decreasing the level of MDA and increasing the level of SOD. Besides, TF3 can activate the expression of Nrf2 in femur, leading to a decrease in ROS in osteoclast.

**Figure 7:**
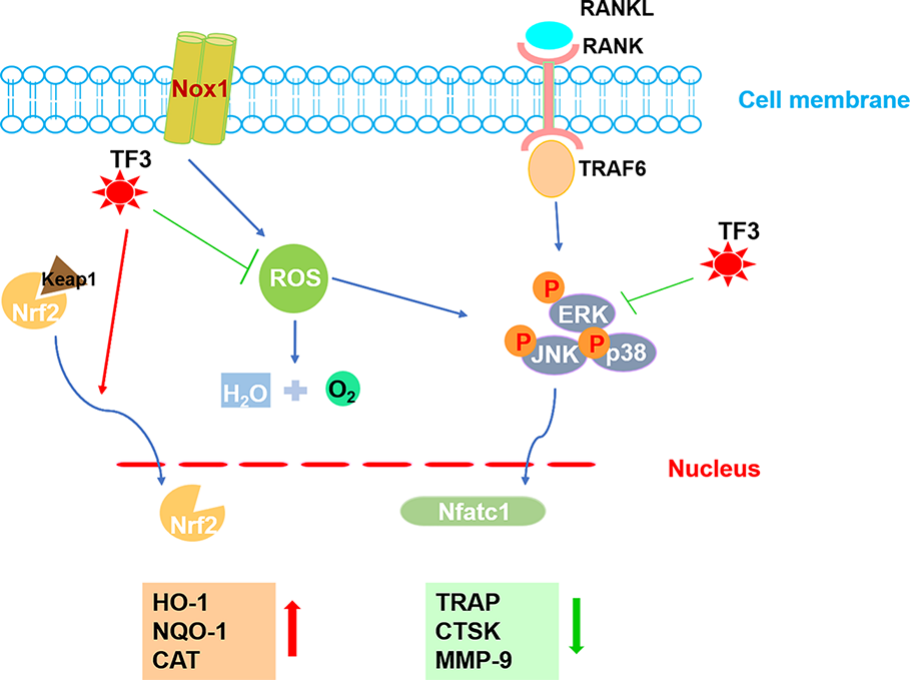
A pattern for the inhibitory effect of TF3 on osteoclastogenesis. Beginning with RANKL binding to RANK, the MAPK pathway is activated, causing the amplification of NFATc1. Several osteoclast-specific genes, such as TRAP, CTSK, and MMP-9 are upregulated. ROS are involved in this process. Our results demonstrated that TF3 inhibits osteoclastogenesis by enhancing the expression of Nrf2-mediated antioxidant enzymes as well as by inhibiting the phosphorylation of the MAPK pathway. The upward-pointing bold arrow shows the enhanced effects of TF3, and the downward-pointing bold arrow displays the inhibitory effects of TF3.

In conclusion, the present study revealed that TF3 raised the level of antioxidant enzymes induced by Nrf2, decreased ROS generation, and inhibited the formation of osteoclasts *in vitro* and *in vivo*. Besides, TF3 also led to damping of the MAPK pathway (**Figure 7**). In addition, TF3 *in vivo* prevented osteoporosis and osteoclastogenesis caused by OVX. Therefore, TF3 may be an appropriate drug for osteoporosis caused by hyperactive osteoclast.

## Data Availability Statement

The raw data supporting the conclusions of this article will be made available by the authors, without undue reservation, to any qualified researcher.

## Ethics Statement

The animal study was reviewed and approved by Animal Ethics Committee of Tongji University.

## Author Contributions

All authors have read and agreed to the published version of the manuscript. ZA: formal analysis, data curation, investigation, writing—original draft. Y’OW: project administration, investigation. MY: resources, visualization. JL: conceptualization, writing—review and editing, funding acquisition. SL: writing—review and editing, funding acquisition, supervision.

## Funding

This work was supported by the Natural Science Foundation of Shanghai (No. 16ZR1439700) and the Science and Technology Commission of Shanghai Municipality (No. 16411960900 and No. 19140904800).

## Conflict of Interest

The authors declare that the research was conducted in the absence of any commercial or financial relationships that could be construed as a potential conflict of interest.
