# Supplementary material for: Theaflavin-3, 3′-Digallate Suppresses RANKL-Induced Osteoclastogenesis and Attenuates Ovariectomy-Induced Bone Loss in Mice
Source: Front Pharmacol. 2020 Jun 29;11:803. doi: 10.3389/fphar.2020.00803 (PMC7336999; doi:10.3389/fphar.2020.00803)
Supplement: Supplementary file 1 [file DataSheet_1.pdf]

Supplementary Material

Table S1 Primer Sequences for RT-qPCR

Gene	Sequence 5′ to 3′	
GAPDH	F: GGGAAGCCCATCACCATCTT	R: GCCTCACCCCATTTGATGTT
TRAP	F: ACGGCTACTTGCGGTTTCA	R: TCCTTGGGAGGCTGGTCTT
MMP-9	F: GCTGACTACGATAAGGACGGCA	R: GCGGCCCTCAAAGATGAACGG
CTSK	F: GAAGAAGACTCACCAGAAGCAG	R: TCCAGGTTATGGGCAGAGATT
Nrf2	F: TCTTGGAGTAAGTCGAGAAGTGT	R: GTTGAAACTGAGCGAAAAAGGC
HO-1	F: GAGATAGAGCGCAACAAGCAG	R: CTTGACCTCAGGTGTCATCTC
CAT	F: CCCCTATTGCCGTTCGATTCT	R: TTCAGGTGAGTCTGTGGGTTT
SOD	F: CAGACCTGCCTTACGACTATGG	R: CTCGGTGGCGTTGAGATTGTT
GPX1	F: AGTCCACCGTGTATGCCTTCT	R: GAGACGCGACATTCTCAATGA

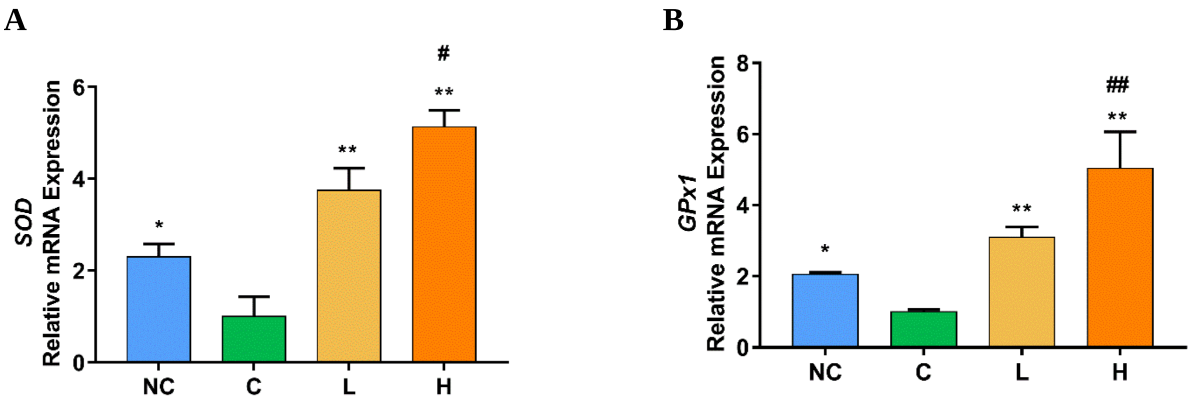

Figure S1 TF3 enhanced the mRNA expression levels of *SOD* and *GPx1*. **(A)**The mRNA expression of *SOD*. **(B)**The mRNA expression of *GPx1*. n = 3, $^{*}P < 0.05$, $^{**}P < 0.01$ compared with C group. $^{\#}P < 0.05$, $^{\#\#}P < 0.01$ compared with L group.

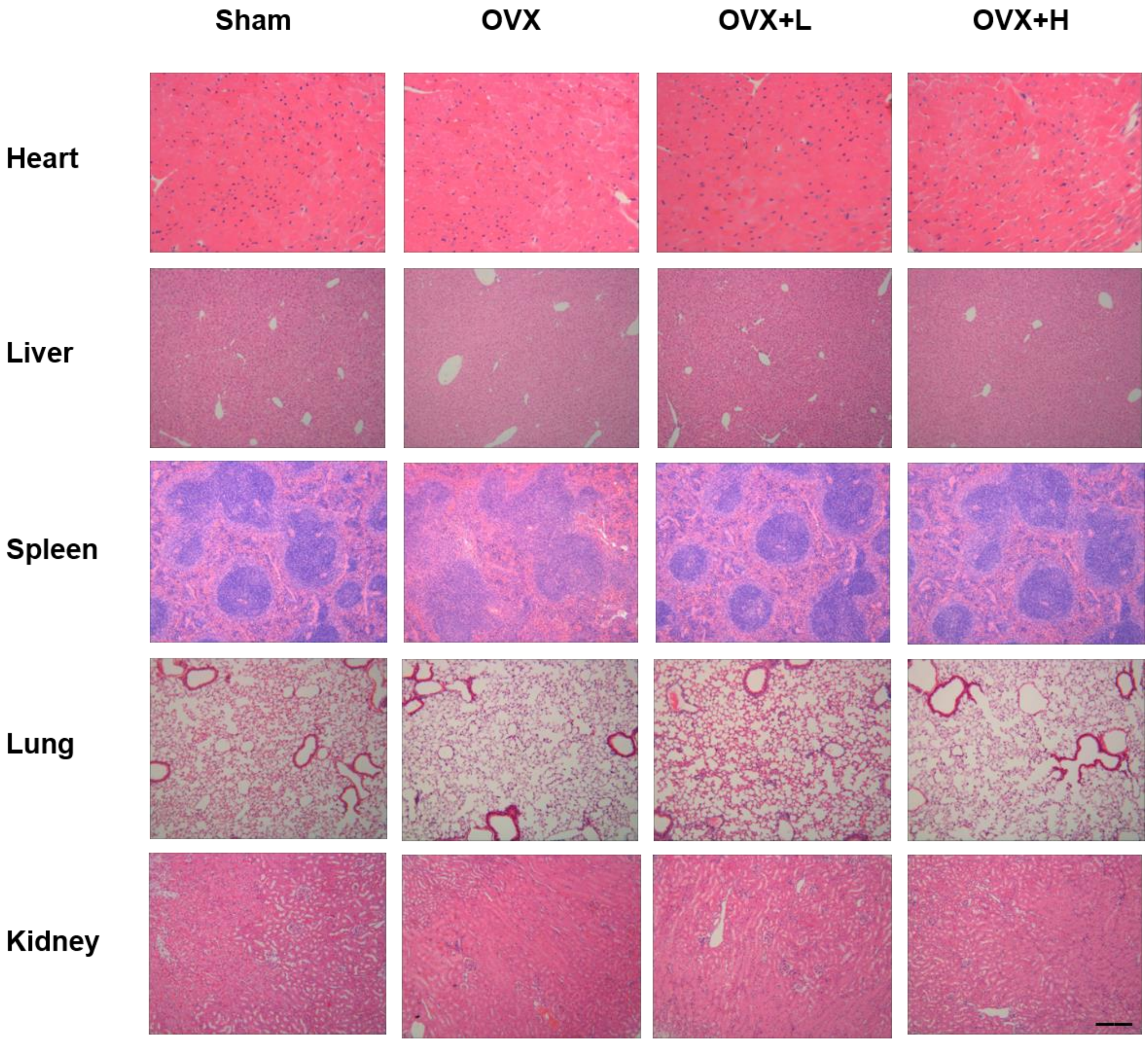

Figure S2 TF3 did not lead to tissue damage in mice. Representative images of H&E staining of heart, liver, spleen, lung kidney of mice. Scale bar = 200 μm.
